# Cytogenetic response of Scots pine (*Pinus sylvestris* Linnaeus, 1753) (Pinaceae) to heavy metals

**DOI:** 10.3897/CompCytogen.v6i1.2017

**Published:** 2012-03-02

**Authors:** Mikhail Vladimirovich Belousov, Olga Sergeyevna Mashkina, Vasily Nikolayevich Popov

**Affiliations:** 1 Voronezh State University, Universitetskaya pl. 1, Voronezh, 394006, Russia

**Keywords:** Scots pine, heavy metals, lead nitrate, zinc nitrate, cupric nitrate, mitosis, chromosomal abnormalities, micronuclei

## Abstract

We studied cytogenetic reactions of Scots pine seedlings to heavy metals – lead, cupric and zinc nitrates applied at concentrations 0.5 to 2000 µM. We determined the range of concentrations of heavy metals that causes mutagenic effect. Lead was found to cause the strongest genotoxicity as manifested by significant increase in the frequency of pathological mitosis, occurrence of fragmentations and agglutinations of chromosomes, various types of bridges, and a significant number of the micronuclei which were absent in the control. Possible cytogenetic mechanisms of the cytotoxic action of heavy metals are discussed.

## Introduction

Over the last decade, heavy metals (HMs) have become the most dangerous and widely spread pollutants of the biosphere and especially soil (Titov et al. 2007). Increase in HMs content is a serious environmental problem. Mining, metallurgy, chemical industry, transport, and widespread use of mineral fertilizers and pesticides are among the major sources of HMs occurrence in the environment. Numerous studies have demonstrated that excessive concentrations of HMs accumulated in plants negatively impact their growth, development and productivity. Apart from the toxic effect, HMs have a mutagenic (genotoxic) effect which is still not sufficiently studied (Steinkellner et al. 1998; Titov et al. 2007; Kulaeva and Tsyganov 2010).

The influence of HMs on plants (usually on grasses and more rarely on trees and shrubs) has been intensively studied by morphological, physiological, and biochemical methods (Bessonova 1991; Chakravarty and Srivastava 1992; Dovgalyuk et al. 2001; Seregin and Ivanov 2001; Fedorkov 2007). There are also data on the influence of HMs at the genetic and cytogenetic levels (Kim et al. 2003; Dobrzeniecka et al. 2011; Vandeligt et al. 2011). However, experimental studies on the cytogenetic responses of conifers to specific metals are not so numerous (Arduini et al. 1994; Prus-Głowacki et al. 2006; Yücel et al. 2008). Scots pine (*Pinus sylvestris* L.) is one of the most common forest forming species in Palaearctic. The investigation of cytogenetic responses of Scots pine seedlings to various HMs will help to estimate the genotoxicity and cytotoxicity of these metals as well as the metal resistance of the plants and develop criteria for selection of tolerant trees for reforestation in anthropogenically polluted areas.

## Material and methods

The seeds of pine trees (*Pinus sylvestris* L.) from the Usmansky forest (the territory of the Voronezh State Biosphere Reserve; sector 80, site 22) were used. The conditions in this site are dry forest (A_1_); the composition is 100% Scots pine. The Usmansky forest is a secondary forest, located along the watersheds of the Voronezh River and the Usman River, Voronezh province, Russia. The quantity of HMs in soil is not excessive (Protasova and Charykova 2011). According to cytogenetic analysis, this pine forest is considered as the benchmark of environmentally safe area (Butorina et al. 2007).

Seeds taken from 10 trees were mixed (in equal amounts from each tree) and placed in Petri dishes on moist filter paper and then germinated at room temperature. In the experimental variants the seeds were pre-soaked in the solutions of lead nitrate Pb(NO_3_)_2_, zinc nitrate hexahydrate Zn(NO_3_)_2_∙6H_2_O, cupric nitrate trihydrate Cu(NO_3_)_2_∙3H_2_O and potassium nitrate KNO_3_ of different concentrations (from 0.5 to 2000 µM) for 18 hours. Then the seeds were germinated in the same solutions in Petri dishes on moist filter paper at a room temperature for 5–7 days. The selected concentrations of HMs are stressful and correspond approximately to: Pb(NO_3_)_2_ – from 3.5 to 14 000 MAC (maximum allowable concentration), Zn(NO_3_)_2_∙6H_2_O – from 3.3 to 13 000 MAC, Cu(NO_3_)_2_∙3H_2_O – from 32 to 128 000 MAC (Dzhuvelikyan 1999). The seeds which were soaked and germinated in distillated water at the same exposure served as the controls. Potassium nitrate was used to exclude the side effect of the nitrate ion impact. The rootlets (reaching lengths of 5–15 mm) of the seedlings from the control and experimental samples were fixed at 9 am and 7 pm (at the peak of the mitotic activity in *Pinus sylvestris* L. in our conditions) in ethanol-acetic mixture (3 parts of 96% ethanol and 1 part of glacial acetic acid). The preparations for cytogenetic analysis stained in aceto-hematoxylin were made according to the technique described earlier (Butorina and Kalaev 2000). More than 20 roots of seedlings (1 rootlet – 1 preparation) were studied for each sample (18 experimental ones and 1 control variant). At minimum 1000 cells were analyzed on each slide. The total number of analyzed cells was more than 380 000. Microphotographs were made with a DCM500 eyepiece digital camera (USB 2.0; WEBBERS MYscope 500 M).

The following parameters were revealed: 1) the mitotic activity of meristematic tissue (which was estimated by the mitotic index (MI)) calculated as a percentage of the number of dividing cells in prophase, meta-, ana-and telophase of mitosis to the total number of counted cells), 2) the percentage of cells in each mitosis phase to determine the duration of these phases, 3) the frequency and spectrum (types) of mitotic abnormalities (MPs) (the frequency was calculated as a ratio of the number of cells with abnormalities in the meta-, ana-, telophase of the mitosis to the total number of dividing cells viewed in %, spectrum of MPs represented as a percentage of each type of pathology to the total number of pathological mitoses), 4) the presence and frequency of cells with micronuclei was calculated as the percentage of cells with micronuclei to the total number of interphase cells counted, and 5) the proportion of cells with *n* number of nucleoli in interphase cells. The number of nucleoli was counted in 500–600 interphase cells for each preparation.

Statistical processing of the data was performed with the help of the statistical program package Stadia and Statistica. The procedure of grouping the data and their treatment are described in the work of Kulaichev (2006). The experimental samples were compared according to the mitotic activity and nucleolar characteristics using Student’s t-test. The comparison of samples in terms of their MPs was carried out using van der Waerden rank X-test, because this feature does not follow the normal distribution. The normality check of distribution was performed using the chi-square test. The influence of the “type of metal” factor was determined using Kruskall-Wallis nonparametric univariate analysis of variance test.

## Results and discussion

Variation of cytogenetic parameters in the root meristem of Scots pine seedlings from the control and experimental samples is presented in Table 1.

*Mitotic activity.* Mitotic activity determines growth energy. Since the mitotic index (MI) is a rather stable parameter at a given time, its change may reflect a mutagenic influence of environmental factors on studied objects (Butorina and Kalaev 2000).

The mitotic index decreased significantly in the seedlings of experimental samples compared with the control under exposure to all three HM salts (lead, zinc and cupric nitrate) (Table 1). However, the decrease did not exceed 1.5% and the MI was in the narrow corridor of 6.0–6.7% regardless of the concentration of HMs. When the experimental samples were compared, the differences were observed only at extreme concentrations. We suppose that a slight decrease in the MI was caused by an inhibition of the existing dividing cells at different stages of mitosis as a response to HMs.

**Figure 1.**
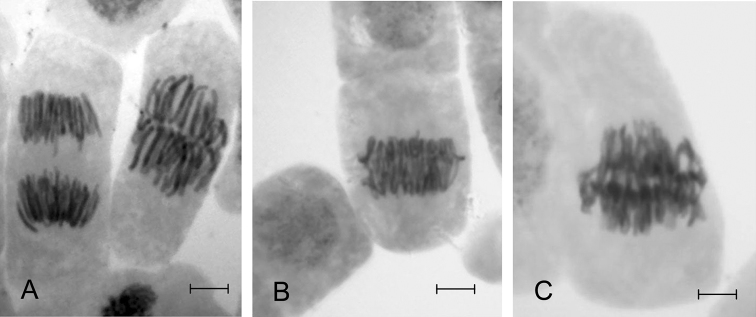
Cells of the root meristem of Scots pine at metaphase and anaphase of mitosis **A** and at the transitional stage of meta-anaphase **B** and **C**. Bar = 10 µm.

A comparison of the frequency of cells at different stages of mitosis revealed that under conditions of stress (experimental samples) there was an increase in the proportion of cells at metaphase, and especially the metaphase-anaphase transition (meta-anaphase) (Fig. 1), as illustrated by the influence of lead nitrate (Table 2). The inhibition of mitosis manifested in a significant (8.5 times for Pb; 7.8 times for Zn; 11.6 times for Cu) increase in the proportion of cells at the intermediate stage of meta-anaphase could be a result of blocking microtubule polymerization as was noted by different authors (Rieder and Palazzo 1992; Wierzbicka 1994; Seoane and Dulout 2001; Thier et al. 2005). For example, it was shown that lead reacts with 2 out of the 18 thiol groups in tubulin dimers of non-polymerized microtubules as well as in already formed ones (Faulstich et al. 1984; Klapheck et al. 1995).

**Table 1.** Average cytogenetic parameters of the root meristem of Scots pine resulting from various exposure concentrations of heavy metals (lead, zinc and cupric nitrates) and potassium nitrate. Differences from the control significant at: ** *P* < 0.01, *** *P* < 0.001.

Cytogenetic<br/> parameter,%	Control	0.5 µM	5 µM	50 µM	500 µM	1000 µM	2000 µM
Lead nitrate							
MI	7.5±01	7.4±0,1	6.6±0.1***	6.4±0.1***	6.1±0.1***	6.1±0.1***	6.0±0.2***
MPs	0.4±0.2	0.5±0.2	4.4±0.4***	5.8±0.3***	7.4±0.3***	8.2±0.4***	11.1±0.4***
micronuclei	0	0.01±0.005	0.05±0.012	0.13±0.014	0.21±0.014	0.31±0.015	0.43±0.025
Zinc nitrate							
MI	7.5±0.1	7.4±0.1	6.8±0.1**	6.7±0.1***	6.6±0.1***	6.6±0.1***	6.4±0.2***
MPs	0.4±0.2	0.4±0.2	3.2±0.4***	5.1±0.5***	5.5±0.4***	7.1±0.4***	9.5±0.5***
micronuclei	0	0	0.02±0.005	0.05±0.006	0.11±0.012	0.19±0.013	0.23±0.015
Cupric nitrate							
MI	7.5±0.1	7.3±0.1	6.7±0.1***	6.6±0.1***	6.6±0.1***	6.5±0.1***	6.1±0.2***
MPs	0.4±0.2	0.5±0.2	2.3±0.4***	3.7±0.4***	4.7±0.4***	5.9±0.4***	6.3±0.5***
micronuclei	0	0	0.01±0.006	0.04±0.014	0.11±0.014	0.18±0.016	0.25±0.016
Potassium nitrate							
MI	7.5±0.1	7.4±0.1	6.4±0.1***	6.1±0.1***	6.1±0.1***	6.2±0.1***	6.6±0.2**
MPs	0.4±0.2	0.3±0.1	0.4±0.2	0.4±0.2	0.5±0.3	0.3±0.1	0.4±0.2
micronuclei	0	0	0	0	0.02±0.009	0.01±0.006	0.04±0.014

**Table 2.** A proportion of cells in % at each stage of mitosis in the root meristem of Scots pine subjected to Pb nitrate. Significance (from control): ** *P* < 0.01, *** *P* < 0.001.<br/>

**Variant**	**Mitosis stage**				
	**Prophase**	**Metaphase**	**Meta-anaphase**	**Anaphase**	**Telophase**
Control	10.4±0.5	30.2±0.9	1.2±0.2	28.4±0.5	29.8±0.4
0.5 µM	9.8±0.4	31.2±1.2	1.3±0.2	27.6±0.7	30.1±0.4
5 µM	10.2±0.5	35.2±1.3***	5.3±0.3***	22.3±1.3***	27.0±1.3**
50 µM	6.6±0.3***	35.9±0.9***	6.8±0.3***	25.6±0.7***	25.1±0.7***
500 µM	9.9±0.6	34.4±0.9***	8.8±0.4***	21.3±0.8***	25.6±0.8***
1000 µM	12.0±0.7***	32.1±1.1**	9.1±0.2***	22.3±1.2***	24.5±0.7***
2000 µM	13.8±0.9***	31.0±1.0	10.2±0.4***	20.9±1.1***	24.1±0.8***

The change of the duration of cells’ passage through the stages of mitosis under the influence of HMs may be associated with the checkpoint activation. In cell cycle, transitions from G_1_ cells to S-phase from G_2 _to mitosis and metaphase to anaphase are viewed as critical stages in which a temporary stop of the cell cycle and check of the genetic material’s integrity take place. Therefore, the “control point” of the cell cycle is a mechanism that protects the dividing cells against lethal mitosis by activation of repair systems for DNA damage or self-destruction of heavily damaged cells via apoptosis. Thus, it is possible to consider the change in duration of cells’ passage in mitosis (an increase in the number of cells in metaphase and meta-anaphase) as one of the mechanisms of adaptation to stressors and maintenance of homeostasis in cell populations of Scots pine seedlings under the influence of Pb, Zn and Cu.

**Figure 2.**
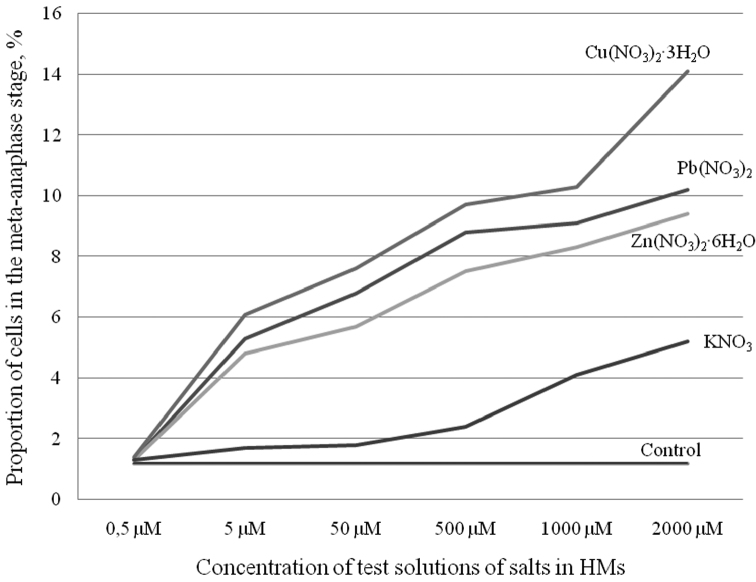
Average percentage of cells at the meta-anaphase stage by concentration of selected salts (lead, zinc, cupric and potassium nitrates) in comparison with the control.

The obtained data on the impact of equal concentrations of various HMs salts on the mitotic activity of root meristem cells allow us to estimate their cytotoxicity. For example, although the decrease in the MI was similar (Table 1) for all HMs salts, the numbers of cells in meta-anaphase were significantly different (Fig. 2). The most toxic under our experimental conditions was Cu; it was followed by Pb and Zn, in agreement with published data (Fargašová 1998; Montvydienė and Marčiulionienė 2004). Thus, the cytotoxic response of Scots pine seedlings to HMs can be presented from the strongest to the weakest as follows: Cu > Pb > Zn.

*Mitotic pathologies and the micronucleus test.* It has been shown for Scots pine that the level and spectrum of mitotic pathologies (MPs), the frequency of cells with micronuclei and the number of nucleoli in the cell are the most sensitive cytogenetic parameters to the anthropogenic pollution, objectively reflecting the state of their genetic system (Butorina and Kalaev 2000).

A significant increase of MPs was found for all tested salts of HMs (lead, zinc and cupric nitrates) starting from the concentration of 5 µM (selected as a threshold). This concentration corresponds to 35 MAC (maximum allowable concentration) for Pb, 33 MAC for Zn and 320 MAC for Cu. The usage of so high concentration in our experiment is explained by the short duration of HMs infulence. Typically, the value of MAC on HMs-polluted territories is lower in natural conditions, but it can come up to 35 MAC for Pb (Dzhuvelikyan 1999). In anthropogenic conditions plants are chronically influenced by HMs so the accumulation of pollutant in the plant tissue should be considered. Our research on the reaction of Scots pine experiencing a chronic combined effect of the influence of pollutants including HMs in the district of the Novolipetsk metallurgic factory revealed substantial changes of cytogenetic parameters compared to those of controls on ecologically favorable territory (Mashkina et al. 2009). There are data (Dobrzeniecka et al. 2011) that genetic variation of *Picea mariana* (Miller, 1768) defined with ISSR markers depends on the degree of moistening of the soil containing the HMs group. The genetic variation was higher in the population of *Picea mariana* growing on the wet lands as compared to those growing on the dry land. But cytological analysis of black spruce seeds from metal-contaminated and uncontaminated areas showed normal mitotic behavior during prophase, metaphase, anaphase, and telophase. There is no research on the influence of the specific HMs on the mitosis under field conditions.

With increase in HMs concentration the frequency of the MPs increased. Starting with the concentration 50 µM for Pb and Zn, the average value of the MPs exceeded normal values of spontaneous mutation level in central Russia by up to 5% (Butorina et al. 2007). Moreover, the sensitivity of the parameter “MPs frequency” was significantly higher than that of MI. The changes (comparing to control) were 11–27 fold (depending on the concentration) for Pb, 8–23 fold for Zn and 5–15 fold for Cu, whereas the MI changes were minimal, and the cell proportion at the stage of meta-anaphase were 11 times greater (maximum for Cu) (Table 1).

**Figure 3.**
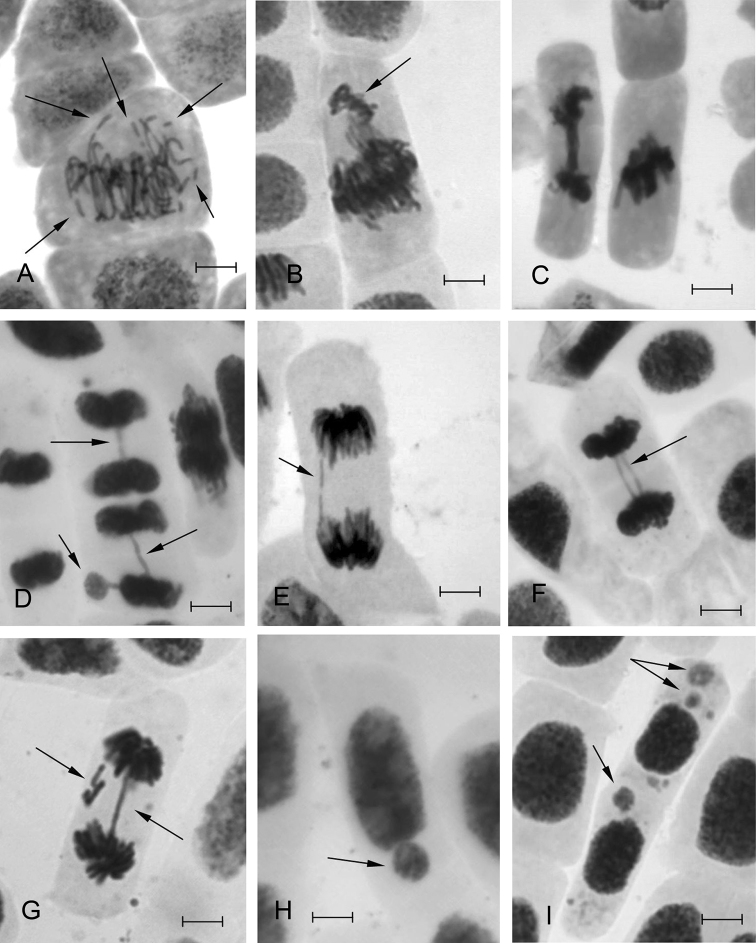
Types of mitotic pathologies and micronuclei in interphase cells found in the root meristem of Scots pine seedlings under exposure to HMs: **A** chromosome fragmentation in metaphase **B** chromosome isolation at meta-anaphase **C** multiple bridge and chromosome agglutination **D** bridge and micronucleus **E** broken bridge **F** double bridge **G** bridge and lagging chromosome fragments **H, I** micronuclei. Bar = 10 µm.

The spectrum of MPs in Scots pine seedlings of experimental samples was manifested by the same abnormalities as identified in control (chromosome segregation and lagging in anaphase, single bridges in ana-telophase) as well as by the new ones such as chromosome fragments in prophase, metaphase, anaphase, chromosome agglutination; chromosome segregation in metaphase and anaphase; different types of bridges (single or multiple, broken bridges, bridge and chromosome fragments, bridge and chromosome lagging etc.) (Fig. 3). The predominance of the lagged chromosomes and marginalized groups of chromosomes observed in the whole spectrum (up to 51.6% for lead) could be due to the inhibited spindle caused by HMs ions. It could also be one of the reasons for a significant number of micronuclei. Similar abnormalities in the control were apparently repaired as micronuclei were not identified. The emergence of chromosome fragments in experimental samples and various types of complex bridges, which totaled up to 48.4% (for Pb), indicate increased levels of mutation (chromosomal rearrangements) under influence of HMs.

**Figure 4.**
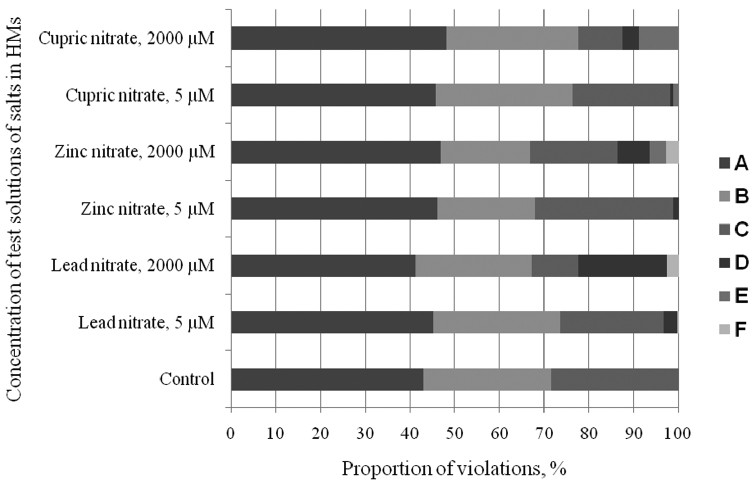
MPs spectrum in cells of the root meristem of Scots pine treated with salts of different concentration: **A** chromosome segregation in metaphase and anaphase **B** bridges **C** lagged chromosomes in anaphase **D** chromosome fragmentation **E** chromosome agglutination **F** multiple violations.

The tested HMs generally caused similar cytogenetic response. Nevertheless, we noticed some specificity of the induced abnormalities. For example, Pb to a greater extent (from 0.4 to 19.8%) caused an appearance of chromosome fragments in prophase and metaphase. Zn had the widest range of induced MPs, including chromosome fragmentation (from 0.4 to 7.3%) and agglutination (0 to 3.6%) and multiple bridges. Cu mainly caused agglutination (0 to 8.8%) and less frequently chromosome fragmentation (from 0 to 3.7%) (Fig. 4).

The differences between the experimental variations were particularly clearly observed in the frequency of occurrence of micronuclei in interphase cells (Table 1). Micronuclei were not identified in the controls. The maximum quantity of micronuclei was observed for Pb (from 0.01 to 0.43% depending on the concentration used), whereas it was lower and almost identical for Zn and Cu (0-0.23% for Zn and 0-0.25% for Cu). The presence of micronuclei indicates unrepaired damage of chromosomal material. It leads to cytogenetic instability in cell populations (Ilyinskikh et al. 1998, Mashkina et al. 2009). Thus, the inhibitory effect of HMs was extended to DNA repair enzymes, as proved by a significant increase in the number of cells containing DNA fragments, bridges, and micronuclei.

The seedlings treated with potassium nitrate showed no significant differences in MP frequency compared with the control (Table 1). Micronuclei were detected only at extreme concentrations (500, 1000 and 2000 µM) and their number was negligible (0.02-0.04%), which excludes the impact of nitrate-ion present in all three salts of HMs (Pb, Zn and Cu).

According to the results Kruskall-Wallis nonparametric univariate analysis of variance test, lead had the greatest impact on the frequency of MPs (Fig. 4A) and the number of cells with micronuclei (Fig. 4B ). The impact of Zn and Cu was not so great. The observed differences were significant at *P* < 0.001. Thus, according to the data on the frequency of micronuclei and MPs, the mutagenic response of Scots pine seedlings to HMs can be presented from the strongest to the weakest as follows: Pb > Zn > Cu.

It was shown (Evseeva et al. 2005) that the toxic and mutagenic action of HMs is based on their ability to bind with amino acids and proteins. Metal ions in enzymes can be also substituted by HMs. HMs induce the formation of reactive oxygen species, protein denaturation, leading to the disruption of cellular metabolism, the processes that cause single breaks of DNA, which is a signal to a change in gene expression. Moreover, the mutagenic effect of HMs detected by the frequency of chromosome aberrations appears at lower concentrations than their cytotoxic effect.

It is well known that Cu and Zn are trace elements essential for cell metabolism, but their excess of the normal physiological level results to a negative impact. Several mechanisms of HM detoxification (including Cu and Zn) that ensure the maintenance of cellular homeostasis and metal resistance increase in plants have been described in a number of papers. These include an immobilization of the cell wall (Zornoza et al. 2002), formation of complexes with chelators (Sanjaya et al. 2008), synthesis of stress proteins (Clemens 2001), synthesis of metallothioneins and phytochelatins (Zhigang et al. 2006; DalCorso et al. 2008; Prévéral et al. 2009). Non-specific reactions to the action of HMs include increased activity of antioxidant enzymes (including peroxidase) that neutralize free radicals and peroxides formed as a result of oxidative stress, rendering the damaging effect on cells (Kuznetsov 2006). Pb is not necessary for living organisms and its cellular metabolism is still poorly studied. Apparently, Pb can undergo partial detoxification as described above because of some similarity (to Ca, for example) of its physical and chemical properties (mainly valency).

The experiments with Scots pine showed (Belousov and Zemlyanukhina 2011) that cytogenetic parameters (the meta-anaphases proportion, the MPs level and spectrum, the micronuclei frequency) were more sensitive to Pb than the activity of a number of respiratory enzymes (including peroxidase – a marker of stress and adaptation), since the changes in the activity of the latter occurred at higher concentrations (500 µM) in contrast to the cytogenetic effects (starting at 5 µM).

*Nucleolar activity*. The role of the nucleolus in metabolic processes is due to its involvement in the biosynthesis of cell rRNA which is necessary for protein biosynthesis. It is known that nucleolar activity in pines can vary within wide limits manifested by the varying number of nucleoli (1 to 12 nucleoli in one nucleus). The number of nucleoli increases under extreme conditions (Muratova 1999, Haydarova and Kalashnik 1999, Butorina and Kalaev 2000). Cells with 3–5 nucleoli (mostly cells with 4 nucleoli) dominated in the control. It is normal for Scots pine (Butorina et al. 2007) and it indicates the predominant activity of nucleolar organizers of two or three pairs of chromosomes. In all experimental samples treated with HMs cells with 5–8 nucleoli (with maximum number of cells containing 6 nucleoli) prevailed. Moreover, the number of cells with the maximum number of nucleoli (10–12 nucleoli in the nucleus) significantly increased compared with the controls (3–5 folds increase, on average). This may be an indicator of metabolic activity enhancement (activation of rRNA genes, ribosomes and protein synthesis) under the influence of HMs as a regulatory mechanism to facilitate the increased protein metabolism in seedlings with MPs increased frequency.

## Conclusion

The presented data show the high genotoxic effect of zinc, cupric and, especially, lead nitrates on Scots pine seedlings. With an increase in HMs concentration (from 5 µM), there were significant changes in cytogenetic parameters. 1) There was a marked inhibition of mitosis manifested in a significant increase in the number of cells in metaphase and intermediate meta-anaphase, which may be caused by blocking the polymerization of tubulin spindle microtubules. However, a decrease in mitotic activity may provide additional time to repair damage (small defects) of chromosomal material in the transition of cells through the “checkpoint” cell cycle. 2) The rate (up to 5–27 fold depending on the concentration of HMs) increased and the spectrum of MPs expanded. Specific chromosome damage, not observed in controls appeared, such as a large number of chromosome fragments (up to 19.8%) and agglutination (8.8%) and a significant amount of micronuclei in interphase cells (up to 0.43%) which indicated the inhibiting impact of HMs on the enzymes of DNA repair systems. 3) The cytogenetic adaptive reactions of Scots pine to the action of HMs included increased cell metabolism (activation of ribosomal rRNA genes, as well as the synthesis of additional, likely stress proteins) due to an increase in cells with the maximum number of nucleoli (10–12) in the nucleus. 4) The data on MPs level and the micronuclei frequency indicate the strength of genotoxic (mutagenic) impact of HMs affecting Scots pine seedlings germination as Pb > Zn > Cu. We show that the most sensitive and suitable for assessing HMs genotoxicity in the early stages of development are the PM level and spectrum, the number of cells with micronuclei and the nucleolar activity.
